# Serological Positivity against Selected *Flaviviruses* and *Alphaviruses* in Free-Ranging Bats and Birds from Costa Rica Evidence Exposure to Arboviruses Seldom Reported Locally in Humans

**DOI:** 10.3390/v14010093

**Published:** 2022-01-06

**Authors:** Daniel Felipe Barrantes Murillo, Marta Piche-Ovares, José Carlos Gamboa-Solano, Luis Mario Romero, Claudio Soto-Garita, Alejandro Alfaro-Alarcón, Eugenia Corrales-Aguilar

**Affiliations:** 1 Pathology Department, Universidad Nacional Costa Rica, Heredia 40104, Costa Rica; dfb0014@auburn.edu (D.F.B.M.); luis.romero.vega@una.cr (L.M.R.); alejandro.alfaro.alarcon@una.cr (A.A.-A.); 2 Virology-CIET (Research Center for Tropical Diseases), Faculty of Microbiology, University of Costa Rica, San Jose 11501, Costa Rica; maria.piche.ovares@una.ac.cr (M.P.-O.); jose.gamboasolano@ucr.ac.cr (J.C.G.-S.); csoto@inciensa.sa.cr (C.S.-G.); 3 Department of Pathobiology, College of Veterinary Medicine, Auburn University, Auburn, AL 6832, USA; 4 PIET (Tropical Disease Research Program), Department of Virology, School of Veterinary Medicine, Universidad Nacional Costa Rica, Heredia 40104, Costa Rica

**Keywords:** arbovirus, bats, birds, wildlife, Costa Rica

## Abstract

Arboviruses have two ecological transmission cycles: sylvatic and urban. For some, the sylvatic cycle has not been thoroughly described in America. To study the role of wildlife in a putative sylvatic cycle, we sampled free-ranging bats and birds in two arbovirus endemic locations and analyzed them using molecular, serological, and histological methods. No current infection was detected, and no significant arbovirus-associated histological changes were observed. Neutralizing antibodies were detected against selected arboviruses. In bats, positivity in 34.95% for DENV-1, 16.26% for DENV-2, 5.69% for DENV-3, 4.87% for DENV-4, 2.43% for WNV, 4.87% for SLEV, 0.81% for YFV, 7.31% for EEEV, and 0.81% for VEEV was found. Antibodies against ZIKV were not detected. In birds, PRNT results were positive against WNV in 0.80%, SLEV in 5.64%, EEEV in 8.4%, and VEEV in 5.63%. An additional retrospective PRNT analysis was performed using bat samples from three additional DENV endemic sites resulting in a 3.27% prevalence for WNV and 1.63% for SLEV. Interestingly, one sample resulted unequivocally WNV positive confirmed by serum titration. These results suggest that free-ranging bats and birds are exposed to not currently reported hyperendemic-human infecting *Flavivirus* and *Alphavirus*; however, their role as reservoirs or hosts is still undetermined.

## 1. Introduction

Arboviruses are zoonotic infectious agents transmitted by hematophagous vectors [1]. More than 135 different types of arbovirus are responsible for clinical infections in humans [1]. Most of these agents are RNA viruses belonging to different genera such as Flaviviruses and Alphaviruses [1]. Flaviviruses (family *Flaviviridae*) such as Dengue virus (DENV), Zika virus (ZIKV), West Nile virus (WNV), Saint Louis encephalitis virus (SLEV), and Yellow Fever virus (YFV), have a positive-sense single-stranded RNA genome of approximately 10.8 kb, which encodes for three structural proteins (E (envelope), C (Capsid), PrM (pre-membrane)) and seven non-structural proteins (NS) (NS1, NS2a, NS2b, NS3, NS4a, NS4b, and NS5) [2,3]. Alphaviruses, specifically the ones that belong to the family *Togaviridae*, such as Eastern Equine Encephalitis virus (EEEV) and Venezuelan Equine Encephalitis virus (VEEV), have a positive-sense single-stranded RNA genome of approximately 11.8 kb, which encodes four non-structural proteins (nsP1-4) and five structural proteins (C, E3, E2, 6K, and E1) [4,5,6].

Some arboviruses are maintained in nature through two different transmission cycles: sylvatic and urban [7]. DENV, YFV, ZIKV, and CHIKV have an exclusive urban cycle in America; on the contrary, WNV, SLEV, EEEV, and VEEV have an enzootic sylvatic with some epizootic cycles’ events [8,9]. In Costa Rica, clinical infections caused by DENV, CHIKV, and ZIKV in humans have been reported circulating every year in a sustained manner [10,11,12]. Additionally, there is serological evidence of SLEV, VEEV, EEEV, and YFV in some wild and domestic animals [13,14,15], and molecular evidence of other arboviruses as well [16]. Thus, Costa Rica is a hyperendemic country for the presence of arbovirus, and there is a continuous increasing interest in the search for possible reservoirs in wildlife. For example, for VEEV, rodents and marsupials have been identified as reservoirs [17]; however, the focus of this research was exclusively on bats and birds. Costa Rica is endemic for some arbovirus such as DENV, ZIKV, and VEEV [12,18,19]. Furthermore, evidence of the circulation of other arboviruses such as WNV, SLEV, and EEEV was found in different mammalian species in Costa Rica, including sloths and horses [13,20]. Therefore, the co-circulation of different arbovirus in this country in many species is very likely.

Bats and birds have a wide distribution and diversity in Costa Rica. Bats represent 45% of the total species of mammals in Costa Rica and encompass 115 different species belonging to nine different families [21]. Costa Rica also possesses an extraordinary diversity of birds; over 922 different species have been reported in the territory, including also migratory birds [22].

For some arboviruses, birds are involved in the sylvatic cycle [23]. Furthermore, bats have also been implicated in maintaining arboviruses in the wild, though inconclusively [24].

To study the role of bats and birds in a putative sylvatic cycle in Costa Rica, we sampled free-ranging bats and birds in two arbovirus endemic locations and analyzed them using molecular, serological, and histological methods. No current infection was detected, and no significant arbovirus-associated histological changes were observed, though neutralizing antibodies were detected against several hyperendemic arboviruses but also several non-endemic arboviruses in Costa Rica such as WNV. Our results suggest that free-ranging bats and birds are exposed to non-endemic *Flaviviruses* and *Alphaviruses*; however, their role as reservoirs or hosts is still undetermined.

## 2. Materials and Methods

**Figure 1:**
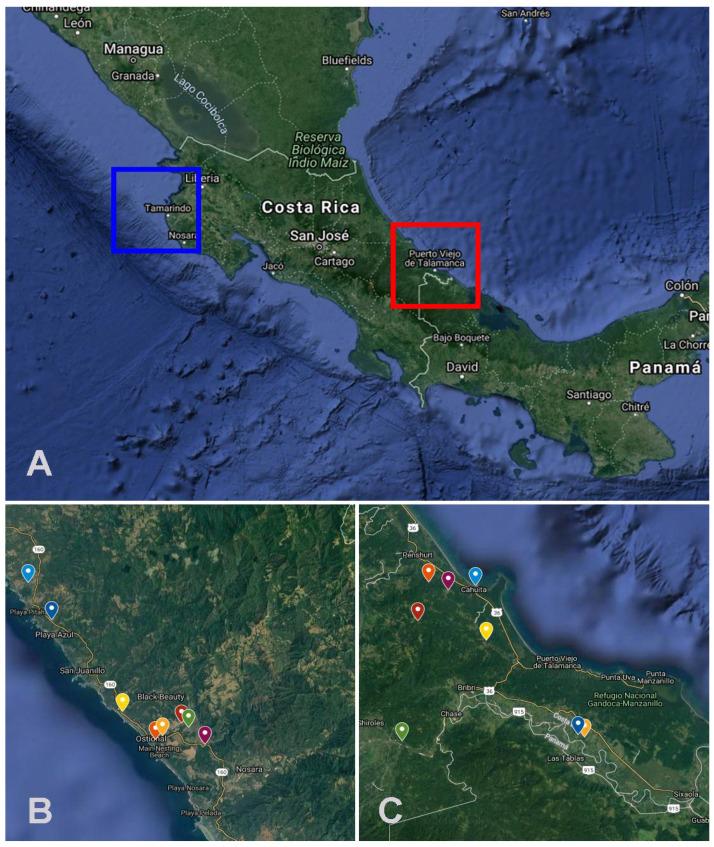
(**A**) Geographic locations of the sampling sites in Costa Rica, the squares indicate Santa Cruz (Northern Pacific) (10°16′00″ N, 85°39′00″ W) and Talamanca (Southern Caribbean) (09°44′28″ N, 82°50′46″ W). (**B**,**C**) Each sampling unit is highlighted in these two regions. Maps Source: Map data ©2021 Imagery ©2021 TerraMetrics and CNES/Airbus, Lansat/Copernicus, Maxar Technologies, US Geological Survey from GoogleMaps.

The sampling was conducted in two regions of Costa Rica during the rainy (more than 250 mm rainfall/month) and the dry seasons (less than 100 mm rainfall/month) [25]. The first region was Santa Cruz, Guanacaste (10°16′00″ N 85°39′00″ W) (Figure 1A,B). The rainy season is between April and November, and the dry season is between December and March. At this site, the sampling process was done between August and November 2017 (during the rainy season) and between February and April 2018 (during the dry season). The second region was Talamanca 09°44′28″ N, 82°50′46″ W) (Figure 1A–C) with one high rainfall season and low rainfall season. The high rainfall season is defined between November and February and between April and September, and the low rainfall season is defined between September and October and between February and March [26]. At this site, the sampling process was done at high rainfall season (July–August 2018) and low rainfall season (September–November 2018).

At each site, eight households (sampling units) were selected and sampled. Birds and bats were collected using mist nets between 15:00–17:00 and 18:00–20:00, respectively. At least five birds and ten bats from each sampling unit were taxonomically identified [27,28], measured, weighed, and euthanized by an intramuscular anesthesia overdose [29] (Supplementary Tables S1–S4). All protocols were approved by the Institutional Committee for the Care and Use of Animals from the University of Costa Rica (CICUA-042-17), Committee of Biodiversity of the University of Costa Rica, and the collection permits from the National System of Conservation Areas (SINAC ACT-PIM-070-17, R-SINAC-PNI-ACLAC-054-2018).

After the euthanasia, a blood sample was taken from the heart. The sample was centrifuged, and the serum was stored at 4 °C until it arrived at the laboratory. Additionally, a complete necropsy procedure was performed in each animal; all tissues were collected and placed in 10% buffered formalin. Furthermore, individual samples of lung, spleen, heart, kidney, liver, brain, eye, knee, intestine, stomach (ventriculus/proventriculus), urinary bladder, and gonads were taken and placed in RNAlater (Life Technologies, Thermo Fisher Scientific Inc., Waltham, MA, USA ) and held at −80 °C. A pool of organs also was taken and placed in RNA later. RNA extraction was performed using TRIzol reagent (Invitrogen, Carlsbad CA, USA), following the manufacturer’s instructions. Complementary DNA (DNAc) was obtained using RevertAidTM RT Kit K#1691 (Thermo Scientific, Waltham, MA, USA) according to the manufacturer’s instructions.

For the identification of viral RNA, a generic RT-PCR using the protocol designed for amplification of partial NS5 segment of Flavivirus [30] and the partial amplification of nsP4 for Alphavirus [31] was performed. The amplicons were analyzed by electrophoresis and interpreted using the equipment QIAxcel Advanced (QIAGEN) and the Software QIAxcel ScreenGel 1.6.0.

A plaque reduction neutralization test (PRNT) assay with chimeric viruses (except for ZIKV, VEE, and YF) was performed with a unique serum dilution (1:10) of each sample to detect neutralizing antibodies; due to the limited amount of sera from each animal, this was the initial sera dilution considered. The average weight of the bats was 24.08 gr (Supplementary Tables S1 and S2), and the average weight of the birds was 32.15 gr (Supplementary Tables S3 and S4). The small-sized of the specimens collected and the use of the blood samples in previous and other experiments limited the possibility of further titration of neutralizing antibodies. Neutralization assays were performed in 96-well, flat-bottomed tissue culture plates because of the limited sera volume. Briefly, each sample was heat-inactivated for 30 min at 56 °C. The serum was diluted at 1.5 in MEM with 2% FBS. Each virus were prepared to an estimated end of 10 UFP/well (WNV (YFV 17D/WNV Flamingo 383-99), DENV 1-4 (YFV 17D/DENV-1 PUO 359, YFV 17D/DENV-2 218, YFV 17D/DENV-3 PaH881/88, YFV 17D/DENV-4 1228), ZIKV (ATCC^®^ VR-748), SLEV (YFV 17D/SLEV CorAn 9124) EEEV-Sindbis, VEEV (TC83 252296), Zika VR 1848 ATCC and YFV 17D). The virus–serum mix was incubated for 1 h at 37 °C. Then, 50 µL of this mixture was added to Vero Cells monolayer previously seeded and incubated for 1 h at 37°C in a 5% CO_2_ atmosphere. After adsorption, the mixture was removed from Vero cells, and 100 µL of MEM with 2% of FBS and 1.5% of carboxymethylcellulose was added (Sigma-Aldrich, St. Louis, MO, USA). Plates were incubated for 72 h at 37 °C in a 5% CO_2_ atmosphere. After the incubation period, MEM containing CMC was removed, and plates were fixed during an hour with formalin (3.7%) and stained with crystal violet (1%). A 90% reduction of foci number relative to the average of the viral control (no sera) was considered positive.

A second retrospective PRNT assay was performed using serum samples from 61 bats previously collected in three different endemic areas: Sarapiquí, Heredia (10°24′20″ N, 84°8′3″ W) Nicoya, Guanacaste (10°9′42″ N, 85°26′48″ W), and Valle Central (9°5′42″ N, 84°8′35″ W) between 2013 and 2014 [32]. PRNT assays were performed using 1:20 dilution, using the same methodology and viruses (except ZIKV, since it was not yet introduced to Costa Rica) described above. These samples from the retrospective study were then categorized as moderately positive (cutoff of ≥50% PRNT on Vero cells) and highly positive (cutoff of ≥90% PRNT on Vero cells). Highly positive samples (>90%) against WNV were submitted for serum titration using serial dilutions from 1:40 to 1:640.

Furthermore, tissue samples fixed in 10% neutral buffered formalin were embedded in paraffin, sectioned at 3 µm, and routinely stained with Hematoxylin and Eosin. Complete histopathological analysis of all tissues was done, characterizing the inflammatory infiltrate, severity, chronicity, and distribution of the lesions. The histopathological analysis aimed to find inflammatory lesions within the central nervous system, joints, reproductive tract, liver, and spleen, previously reported in experimental animal models and the natural viral infection [33,34,35,36,37].

## 3. Results

For this study, a total of 144 bats (75 males and 69 females) representing 5 families and 26 species were collected (Supplementary Tables S1 and S2). A total of 72 individuals were collected in Santa Cruz (42 during the rainy season 2017 and 30 during the dry season 2018). A total of 72 individuals were collected from Talamanca (41 during the high rainfall season and 31 during the low rainfall season in 2018). Due to the limited amount of serum, PRNT 1:10 dilution was performed only in 123 serum samples from bats. Neutralizing antibodies were detected against DENV 1-4, ZIKV, YFV, WNV, SLEV, EEEV, and VEEV. In bats, 34.95% (43/123) for DENV-1, 16.26% (20/123) for DENV-2, 5.69% (7/123) for DENV-3, 4.87% (6/123) for DENV-4, 2.43% (3/123) for WNV, 4.87% (6/123) for SLEV, 0.81% (1/123) for YFV, 7.31% (9/123) for EEEV, and 0.81% (1/123) for VEEV were found as positive. Antibodies to ZIKV were not detected. Cross-reactions among the different types of Flaviviruses and Alphaviruses were present in 39.28% of the positive samples (22/56) (Table 1). A percentage (17.88%) of the bats were exclusively positive (cutoff of ≥90% PRNT on Vero cells and no reaction for other viruses) for DENV-1 (22/123), 3.25% were exclusively positive for DENV-2 (4/123), 0.81% for DENV-3 (1/123), 0.81% for DENV-4 (1/123), 0.81% for WNV (1/123), 3.25% for EEEV (4/123), and 0.81% for VEEV (1/123) (Table 2). No individuals with neutralizing antibodies exclusively against SLEV and YFV were found.

We expanded the PRNT assays in 61 out of 241 bat samples that have previously been tested solely against DENV in one of our previous studies, in which we reported a 21.2% DENV seropositivity in bats [32]. Now, these sera were retrospectively assessed once again but against other *Flaviviruses*. The preliminary 1:20 dilution results showed that 11.4% (7/61) have neutralizing antibodies against WNV and 14.75% (9/61) against SLEV (Table 3). The samples were then categorized as moderately positive (cutoff of ≥50% PRNT) and highly positive (cutoff of ≥90% PRNT). From all the samples analyzed, 19.67% (12/61) were classified as moderately positive, and only 4.91% (3/61) were highly positive against SLEV and WNV. Of the moderately positive samples, 58% (7/12) present serological cross-reaction against at least one serotype of DENV (Table 3). Highly positive samples were tested by further dilution of sera when it was available. Surprisingly and interestingly, a highly positive sample against WNV presented a neutralizing titer of >1:640 for WNV with a titer of <1:40 for SLEV and DENV 1–4. After this reassessment and due to the observed serological cross-reaction between arboviruses, only highly positive samples were then considered truly seropositive for WNV or SLEV. Therefore, overall prevalence exclusively for WNV and SLEV in these bats was 3.27% (2/61) and 1.63% (1/61), respectively. No WNV or SLEV circulation in these areas (Valle central) has been so far reported in humans or wildlife.

A total of 140 birds were collected (89 males and 51 females), representing 37 genera and 43 species (Supplementary Tables S3 and S4). In total, 52 individuals were collected at Santa Cruz (16 during the rainy season in 2017 and 35 during the dry season in 2018); 88 individuals were collected in Talamanca (60 during high rainfall season and 28 during low rainfall season in 2018). Serum neutralization analysis (PRNT 1:10) was performed against WNV and SLEV in 124 serum samples and EEEV and VEEV in 71 serum samples due to sample volume constraints. In birds, PRNT resulted positive against WNV in 0.80% (1/124), SLEV in 5.64% (7/124), EEEV in 8.4% (6/71), and VEEV in 5.63% (4/71). Cross-reactions were also observed in two individuals presenting antibodies against EEEV and VEEV (Table 4).

All RT-PCR analyses performed in both blood and pool tissues collected from bats and birds were negative for the detection of viral RNA from flaviviruses and alphaviruses. Histopathological findings were nonspecific, inflammatory, and degenerative changes frequently associated with the presence of diverse parasites (protozoa, metazoans). Thus, histological analysis of tissues did not show any significant findings related to arboviral infections.

## 4. Discussion and Conclusions

The sylvatic cycle from some arboviruses has not been thoroughly described in tropical regions. Some bird species have been identified as amplifiers of WNV and SLEV in North America [38,39,40,41]. However, Costa Rica lacks information about which species may be implicated in these sylvatic or peri-urban cycles. Nevertheless, even though they represent one of the most abundant mammals and diverse species in many tropical and neotropical areas, bats are still not directly implicated as part of the virus cycle of the most important arboviruses infecting humans such as DENV, ZIKV, and WNV, but they have been described as accidental hosts [24,32]. In many areas, the identification of wildlife susceptible to infection and thus being able to participate as hosts or reservoirs is not fully elucidated, and surely, just identifying exposure does not translate into being part of the virus cycle. Nevertheless, because of the importance of this identification and to further study the role of wildlife in a putative sylvatic cycle in Costa Rica, we sampled free-ranging bats and birds in two arbovirus endemic locations and analyzed them using molecular, serological, and histological methods. No current infection was detected, and no significant arbovirus-associated histological changes were observed. However, neutralizing antibodies were detected against several arboviruses. These results demonstrate that free-ranging bats and birds in Costa Rica are exposed to non-endemic Flaviviruses and Alphaviruses.

PRNT is the gold standard for the serological diagnosis of previous arboviral infection [23]. Using PRNT with chimeric viruses, we demonstrated the presence of antibodies against several Flaviviruses (DENV1-4, SLEV, YFV, WNV) and Alphaviruses (EEEV, VEEV) in bats and birds in Costa Rica. However, as expected, cross-reactions with different Flaviviruses and Alphaviruses were prevalent in our microneutralization assays. Cross-reactions make it difficult to establish the exposure to the etiologic virus unequivocally. This phenomenon is explained by the similarities between the immunodominant epitopes within the family *Flaviviridae* and *Togaviridae* [23]. The envelope structural glycoprotein is the main target for the neutralizing antibodies in *Flaviviridae* and E2 structural protein for *Togaviridae* [42]. To determine against which specific arbovirus the animal was exposed to, dilution titration of the serum is necessary [23]. Unfortunately, the scant amount of serum available in most of our samples did not allow this. However, studies suggest that the serological evidence in a PRNT allows determining that bats were exposed against a given arbovirus in a period between 10 days to months before the analyses [23]. In the retrospective serological study with samples that were preliminary positive for DENV, serum titration allowed the identification and confirmation of a single bat from an urban area (Valle Central) unequivocally positive to WNV (*Molossus sinaloe*) with a titer > 1:640 [43].

On the other hand, viral RNA was not evidenced in the blood or tissues from any of the sampled animals. Because arboviral infections are generally transient, the chance of detecting viral RNA from sera or tissues is extremely low [44]. In this study, we did not detect any active viremia in bats and birds.

This is not the first evidence of arboviruses in bats in Costa Rica. A previous study demonstrated serological prevalence in 21.2% (51/241) and positivity by RT-PCR in 8.8% (28/318) bats for DENV RNA [32]. The retrospective PRNT assays were performed with the samples obtained from this previous project. However, these results contrast with our findings, in which we cannot evidence viral RNA in the animal blood and/or tissues. In the previous report, it was speculated ingestion of mosquitoes was the route of infection since most of the collected samples were from insectivorous animals. Bats as a dead-end host for DENV were then suggested [32]. The majority of the bats collected in this study are frugivorous, and we did not detect any viral RNA in any organ, including intestines. Furthermore, during histological evaluation on the intestines, no evidence of ingested arthropods was observed. Thus, infection through the ingestion of mosquitoes might be unlikely. Mosquito feeding, therefore, seems to be the more plausible route of infection.

In other locations in the American continent, bats have been subjected to extensive evaluations for arbovirus infection with different outcomes. In Brazil, 103 samples from free-ranged bats were analyzed via nested-PCR and hemagglutination inhibition test. All the samples were negative for arbovirus; thus, the authors concluded that bats do not constitute a reservoir for these viruses in the studied areas [45]. In Trinidad, a serological analysis demonstrated a prevalence of 2.9% (11/384) against VEEV using an ELISA in bats [44]. In Colombia, VEEV viral RNA and antigen detection were demonstrated via RT-PCR and immunohistochemistry (IHC) in the brain tissues from two frugivorous bats, suggesting that these species might be hosts for this zoonotic agent [46]. Finally, a new strain of encephalitogenic VEEV (Tonate Virus) was isolated from bats in French Guiana [47]. Neutralizing antibodies against SLEV have been reported in bats from Ohio, USA [48]. WNV has never been reported in bats in Central America [49]; there are only two reports of the presence of neutralizing antibodies against WNV in the USA [50,51]. YFV and ZIKV infection has not been reported so far in bats in America [52]. We found only one bat with neutralizing antibodies against YFV but with a clear cross-reaction against DENV-1, DENV-2, and WNV. Only one of the three bats positive against WNV was positive exclusively and unequivocally to this agent. In the retrospective assay, one *Molossus sinaloe* was confirmed through serum titration (1:640) as seropositive against WNV. Thus, this is the first evidence of WNV exposure in bats in Central America.

This is the first time that SLEV, WNV, EEEV, and VEEV exposure have been demonstrated in wild birds in Costa Rica. Overall, reported seroprevalence against those agents is low in America. A study conducted in Yucatán México revealed a seroprevalence of 4.3% (11/257) against WNV through ELISA [38]. In Trinidad, one study reported a seroprevalence of 1.4% (2/140) against SLEV through the hemagglutinin inhibition test [53]. WNV and SLEV can cross-react [39], and serum titration was not performed. Only one species, *Empidonax virescens* is migratory and has neutralizing antibodies against SLEV and cross-reaction between VEEV and EEEV, suggesting a non-local exposure. None of the species identified in this study has been reported as a reservoir for SLEV and WNV [23]. Therefore, we conclude that some of our sampled birds were exposed to these viruses, but since we did not detect any active viremia, we were not able to identify the putative reservoirs for these viruses in Costa Rica. Thus, further sampling efforts in exemplar numbers and areas must be conducted.

Costa Rica is a hyperendemic country for arboviruses such as DENV, ZIKV, and VEEV. Our results show that not only are these viruses actively circulating, but also other agents such as WNV and SLEV circulate, though silently. Therefore, more studies must be planned to determine the extent of the presence of these viruses in Costa Rica. Alpha- and Flaviviruses not only tend to cross-react serologically among them but also present similar clinical features, so a differential diagnosis must be made. In this case, the PRNT becomes the gold standard to differentiate between the members of each viral family.

Taken together, we can conclude that free-ranging bats and birds are exposed to not currently reported hyperendemic-human infecting *Flaviviruses* such as WNV and some *Alphaviruses*; however, their role as reservoirs or hosts is still undetermined. They may have a role as a dead-end host, but no evidence supports that the sampled species during this study might function as reservoirs.

## Figures and Tables

**Table 1:** Species of bats presenting serological cross-reaction among viruses (*Flaviviruses* and *Alphaviruses*) were analyzed in the PRNT 1:10 dilution.

Species	Sex	Positive Results in PRNT 1:10 (Putative Cross-Reactions)
*Glossophaga soricina*	Female	DENV-1/DENV-3
*Artibeus jamaicensis*	Female	DENV-1 DENV-2
*Artibeus phaeotis*	Female	DENV-1 DENV-2 DENV-4
*Artibeus phaeotis*	Female	DENV-1 DENV-2 DENV-4
*Phyllostomus discolor*	Male	DENV-1 EEEV
*Phyllostomus discolor*	Female	DENV-1 DENV-2 DENV-3 EEEV
*Noctilio albiventris*	Male	DENV-1 EEEV
*Artibeus lituratus*	Female	DENV-1/DENV-2/ DENV-3
*Artibeus jamaicensis*	Male	DENV-1 DENV-2 EEEV
*Artibeus phaeotis*	Male	DENV-1 DENV-2 WNV SLEV
*Carollia perspicillata*	Male	DENV-1 DENV-2
*Artibeus watsoni*	Male	DENV-1 DENV-2 DENV-3 DENV-4
*Artibeus jamaicensis*	Female	DENV-1 SLEV
*Carollia perspicillata*	Male	DENV-1 DENV-2
*Glossophaga soricina*	Female	DENV-1 DENV-2 DENV-3 DENV-4
*Artibeus jamaicensis*	Female	DENV-1 DENV-2 YFV WNV
*Artibeus jamaicensis*	Female	SLEV, EEEV
*Uroderma convexum*	Female	DENV-1 DENV-2 DENV-3 SLEV
*Uroderma convexum*	Female	DENV-1 DENV-2 SLEV
*Carollia perspicillata*	Female	DENV-1 DENV-2
*Artibeus jamaicensis*	Male	DENV-1 DENV-2
*Artibeus jamaicensis*	Male	DENV-1 DENV-4 SLEV

**Table 2:** Species of bats with neutralizing antibodies exclusive (specific) against *Flaviviruses* or *Alphaviruses* at PRNT 1:10 dilution.

Species	Sex	Exclusive Reaction in PRNT 1:10
*Uroderma convexum*	Male	DENV-1
*Artibeus phaeotis*	Male	DENV-1
*Desmodus rotundus*	Female	DENV-1
*Sturnira parvidens*	Female	DENV-1
*Artibeus jamaicensis*	Male	DENV-1
*Chiroderma salvini*	Male	DENV-1
*Ptenorotus mesoamericanus*	Female	DENV-1
*Carollia perspicillata*	Female	DENV-1
*Uroderma convexum*	Male	DENV-1
*Glossophaga soricina*	Male	DENV-1
*Loncophylla robusta*	Female	DENV-1
*Myotis nigricans*	Female	DENV-1
*Phyllostomus discolor*	Male	DENV-1
*Artibeus jamaicensis*	Male	DENV-1
*Glossophaga soricina*	Male	DENV-1
*Carollia perspicillata*	Female	DENV-1
*Carollia perspicillata*	Male	DENV-1
*Artibeus jamaicensis*	Female	DENV-1
*Uroderma convexum*	Male	DENV-1
*Carollia castanea*	Female	DENV-1
*Rhogeessa io*	Female	DENV-1
*Uroderma convexum*	Male	DENV-1
*Glossophaga soricina*	Female	DENV-2
*Artibeus lituratus*	Male	DENV-2
*Artibeus watsoni*	Male	DENV-2
*Glossophaga soricina*	Male	DENV-2
*Artibeus phaeotis*	Female	DENV-3
*Desmodus rotundus*	Female	DENV-4
*Carollia castanea*	Female	WNV
*Carollia perspicillata*	Female	EEEV
*Phyllostomus discolor*	Male	EEEV
*Phyllostomus discolor*	Male	EEEV
*Artibeus jamaicensis*	Male	EEEV
*Carollia perspicillata*	Male	VEEV

**Table 3:** Species of bats categorized as moderately or highly neutralizing samples against SLEV and WNV by PRNT showed cross-reaction with other *Flaviviruses*.

Species	Sex	Cross-Reactions in PRNT 1:20
*Molossus sinaloe*	Female	SLEV, DENV-3 **
*Molossus sinaloe*	Female	WNV, DENV-3 **
*Molossus sinaloe*	Female	SLEV, DENV-3 **
*Molossus sinaloe*	Female	WNV, DENV-3 **
*Molossus sinaloe*	Female	WNV *, DENV-3 **
*Molossus sinaloe*	Male	WNV, SLEV, DENV-2 **, DENV-3 **
*Molossus sinaloe*	Male	SLEV, DENV-3 **
*Molossus sinaloe*	Male	SLEV, DENV-1 **, DENV-2 **, DENV4 **
*Uroderma bilobatum*	Male	WNV, DENV-3 **

* Sample titrated and with antibodies titers against WNV > 1:640; ** data from our previous study already published [24].

**Table 4:** Species of birds seropositive in the PRNT 1:10 dilution against selected arbovirus.

Species	Sex	PRNT 1:10 Result
*Aimophila ruficauda*	Male	EEEV
*Turdus grayi*	Male	SLEV
*Pitangus sulphuratus*	Female	EEEV
*Quiscalus mexicanus*	Female	VEEV
*Icterus pustulatus*	Male	EEEV/VEEV
*Icterus pustulatus*	Male	EEEV
*Campylorhynchus rufinucha*	Female	WNV
*Pitangus sulphuratus*	Male	VEEV
*Crotophaga sulcirostris*	Male	EEEV
*Myozetetes similis*	Male	SLEV
Empidonax virescens *	Male	SLEV/EEEV/VEEV

* Migratory species.

## Supplementary Materials

The following supporting information can be downloaded at: https://www.mdpi.com/article/10.3390/v14010093/s1, Table S1: List of bats collected in Santa Cruz; Table S2: List of bats collected in Talamanca; Table S3: List of birds collected in Santa Cruz; Table S4: List of birds collected in Talamanca.
