# Supplementary material for: Serological Positivity against Selected Flaviviruses and Alphaviruses in Free-Ranging Bats and Birds from Costa Rica Evidence Exposure to Arboviruses Seldom Reported Locally in Humans
Source: Viruses. 2022 Jan 6;14(1):93. doi: 10.3390/v14010093 (PMC8780000; doi:10.3390/v14010093)
Supplement: Supplementary file 1 [file viruses-14-00093-s001.zip › Supplementary Table S1.pdf]

Supplementary Table S1. List of bats collected in Santa Cruz.

**Identification**	**Species**	**Sex**	**Age**	**Reproductive status**	**Weight (g)**	**Collection site Identification**	**Mist Net Location**
MSCA2	*Saccopteryx leptura*	Female	Adult	Inactive	5	CSCA	Barn
MSCA4	*Saccopteryx bilineata*	Male	Adult	Inactive	6	CSCA	Barn
MSCA6	*Glossophaga soricina*	Female	Adult	Inactive	9	CSCA	Forest
MSCA7	*Saccopteryx leptura*	Na	Adult	Inactive	4	CSCA	Barn
MSCA14	*Glossophaga soricina*	Female	Adult	Inactive	11	CSCA	Forest
MSCB1	*Artibeus jamaicensis*	Male	Adult	Inactive	41.5	CSCB	Forest
MSCB2	*Carollia perspicillata*	NA	Adult	Inactive	19	CSCB	Forest
MSCB3	*Noctilio leporinus*	Male	Adult	Inactive	55	CSCB	House
MSCB4	*Noctilio leporinus*	Na	Adult	Inactive	50	CSCB	House
MSCB5	*Artibeus jamaicensis*	Male	Adult	Inactive	21.5	CSCB	House
MSCC1	*Uroderma convexum*	Male	Juvenile	Inactive	9	CSCC	Forest
MSCC2	*Micronycteris hirsuta*	Female	Adult	Nursling	19	CSCC	Forest
MSCC3	*Glossophaga soricina*	Female	Adult	Inactive	8.5	CSCC	Peridomiciliary
MSCC4	*Phyllostomus discolor*	Male	Adult	Inactive	39	CSCC	Forest
MSCC5	*Phyllostomus discolor*	Female	Adult	Pregnant	39	CSCC	Forest
MSCD1	*Artibeus jamaicensis*	Male	Adult	Inactive	39	CSCD	Peridomiciliary
MSCD2	*Rhogeessa tumida*	Female	Adult	Inactive	4.5	CSCD	Peridomiciliary
MSCD3	*Artibeus jamaicensis*	Male	Adult	Inactive	NA	CSCD	Forest
MSCD4	*Glossophaga soricina*	Female	Adult	Inactive	9	CSCD	Forest
MSCE1	*Artibeus phaeotis*	Male	Adult	Inactive	11	CSCE	Forest
MSCF1	*Chiroderma trinitatum*	Female	Adult	Inactive	11	CSCF	Barn
MSCF2	*Myotis nigricans*	Female	Adult	Inactive	4	CSCF	Barn

Identification	Species	Sex	Age	Reproductive status	Weight (g)	Collection site Identification	Mist Net Location
MSCF3	*Desmodus rotundus*	Female	Adult	Inactive	45	CSCF	No Unspecified
MSCF4	*Artibeus phaeotis*	Male	Adult	Inactive	11	CSCF	House
MSCF5	*Desmodus rotundus*	Female	Adult	Inactive	53	CSCF	House
MSCG3	*Saccopteryx leptura*	Female	Adult	Inactive	7	CSCG	Domiciliary
MSCG4	*Artibeus jamaicensis*	Female	Adult	Inactive	46	CSCG	Forest
MSCG5	*Artibeus jamaicensis*	Male	Adult	Inactive	34	CSCG	Forest
MSCG7	*Artibeus jamaicensis*	Male	Adult	Inactive	49	CSCG	Domiciliary
MSCG9	*Sturnira parvidens*	Female	Adult	Inactive	13	CSCG	Forest
MSCG11	*Glossophaga soricina*	Male	Adult	Inactive	17	CSCG	Domiciliary
MSCG12	*Artibeus jamaicensis*	Female	Adult	Inactive	33	CSCG	Forest
MSCG14	*Artibeus jamaicensis*	Female	Adult	Inactive	28	CSCG	Forest
MSCG17	*Glossophaga soricina*	Female	Adult	Inactive	14	CSCG	Forest
MSCH1	*Artibeus jamaicensis*	Male	Adult	Inactive	41	CSCH	Forest
MSCH2	*Sturnira parvidens*	Female	Adult	Pregnant	16	CSCH	Domiciliary
MSCH4	*Sturnira parvidens*	Female	Adult	Pregnant	16	CSCH	Forest
MSCH6	*Artibeus jamaicensis*	Male	Adult	Inactive	37	CSCH	Forest
MSCH8	*Artibeus jamaicensis*	Male	Adult	Inactive	36	CSCH	Forest
MSCH10	*Sturnira parvidens*	Female	Adult	Pregnant	20	CSCH	Forest
MSCH11	*Sturnira parvidens*	Female	Adult	Pregnant	21	CSCH	Forest
MSCI1	*Artibeus jamaicensis*	Male	Adult	Inactive	49	CSCA	River
MSCI2	*Artibeus phaeotis*	Female	Adult	Inactive	12	CSCA	River
MSCI3	*Chiroderma salvini*	Male	Adult	Inactive	16	CSCA	River
MSCI4	*Artibeus phaeotis*	Female	Adult	Pregnant	12	CSCA	Forest
MSCI5	*Sturnira parvidens*	Female	Adult	Pregnant	21	CSCA	Forest

**Identification**	**Species**	**Sex**	**Age**	**Reproductive status**	**Weight (g)**	**Collection site Identification**	**Mist Net Location**
MSCJ1	*Pteronotus mesoamericanus*	Female	Adult	Pregnant	18.5	CSCB	Barn
MSCJ5	*Pteronotus mesoamericanus*	Female	Adult	Pregnant	19	CSCB	Barn
MSCJ7	*Sturnira parvidens*	Female	Adult	Inactive	14	CSCB	Barn
MSCJ9	*Artibeus jamaicensis*	Male	Adult	Inactive	38.5	CSCB	Forest
MSCJ13	*Carollia perspicillata*	Male	Adult	Inactive	19	CSCB	Forest
MSCK1	*Carollia perspicillata*	Female	Adult	Inactive	19	CSCC	Forest
MSCK2	*Rhynchonycteris naso*	Female	Adult	Inactive	4	CSCC	Peridomiciliary
MSCL1	*Hylonycteris underwoodi*	Female	Adult	Inactive	10	CSCD	Forest
MSCL3	*Carollia perspicillata*	Male	Adult	Inactive	18.5	CSCD	Forest
MSCL5	*Rhogeessa tumida*	Female	Adult	Pregnant (twins)	4	CSCD	Peridomiciliary
MSCL7	*Carollia perspicillata*	Female	Adult	Inactive	15	CSCD	Forest
MSCL9	*Desmodus rotundus*	Female	Adult	Inactive	32	CSCD	Forest
MSCM3	*Phyllostomus discolor*	Na	Adult	Inactive	41	CSCE	Forest
MSCM4	*Phyllostomus discolor*	Male	Adult	Inactive	43	CSCE	Forest
MSCM5	*Phyllostomus discolor*	Male	Adult	Inactive	35	CSCE	Forest
MSCM6	*Phyllostomus discolor*	Male	Adult	Inactive	41	CSCE	Forest
MSCM7	*Phyllostomus discolor*	Female	Adult	Inactive	37.5	CSCE	Forest
MSCN1	*Artibeus lituratus*	Female	Adult	Pregnant	66	CSCF	Forest
MSCN2	*Pteronotus mesoamericanusi*	Female	Adult	Inactive	18	CSCF	Forest
MSCN3	*Noctilio albiventris*	Male	Adult	Inactive	36	CSCF	Forest
MSCN4	*Artibeus lituratus*	Female	Adult	Pregnant	62	CSCF	Forest
MSCN6	*Glossophaga soricina*	Male	Adult	Inactive	9	CSCF	Forest
MSCO1	*Artibeus jamaicensis*	Male	Adult	Inactive	41	CSCH	Forest
MSCO2	*Artibeus jamaicensis*	Female	Adult	Pregnant	43	CSCH	Forest

**Identification**	**Species**	**Sex**	**Age**	**Reproductive status**	**Weight (g)**	**Collection site Identification**	**Mist Net Location**
MSCO4	*Artibeus phaeotis*	Male	Adult	Inactive	10	CSCH	House
MSCO6	*Carollia perspicillata*	Male	Adult	Inactive	17.5	CSCH	Forest
MSCO7	*Artibeus lituratus*	Male	Adult	Inactive	61	CSCH	Forest
