# Supplementary material for: Serological Positivity against Selected Flaviviruses and Alphaviruses in Free-Ranging Bats and Birds from Costa Rica Evidence Exposure to Arboviruses Seldom Reported Locally in Humans
Source: Viruses. 2022 Jan 6;14(1):93. doi: 10.3390/v14010093 (PMC8780000; doi:10.3390/v14010093)
Supplement: Supplementary file 1 [file viruses-14-00093-s001.zip › Supplementary Table S2.pdf]

Supplementary Table S2. List of bats collected in Talamanca.

Identification	Species	Sex	Age	Reproductive status	Weight (g)	Collection site Identification	Mist Net Location
MTAA1	*Uroderma convexum*	Male	Adult	Inactive	15	CTAA	Peridomiciliary
MTAB1	*Noctilio leporinus*	Male	Adult	Inactive	66	CTAB	Forest
MTAB2	*Artibeus watsoni*	Male	Adult	Inactive	10.5	CTAB	Peridomiciliary
MTAB4	*Artibeus watsoni*	Male	Adult	Inactive	10.5	CTAB	Forest
MTAB5	*Carollia perspicillata*	Male	Adult	Inactive	18.5	CTAB	Forest
MTAB7	*Artibeus jamaicensis*	Female	Adult	Pregnant	57	CTAB	Forest
MTAB8	*Carollia perspicillata*	Male	Adult	Inactive	18	CTAB	Forest
MTAB10	*Artibeus jamaicensis*	Male	Adult	Inactive	46	CTAB	Forest
MTAB11	*Uroderma convexum*	Male	Adult	Inactive	14	CTAB	Forest
MTAB13	*Carollia perspicillata*	Female	Adult	Pregnant	23	CTAB	Forest
MTAC1	*Uroderma convexum*	Female	Adult	Inactive	15	CTAC	Forest
MTAC2	*Glossophaga soricina*	Male	Adult	Inactive	9.5	CTAC	Peridomiciliary
MTAC3	*Glossophaga soricina*	Male	Adult	Inactive	9	CTAC	Forest
MTAC4	*Glossophaga soricina*	Female	Adult	Pregnant	12	CTAC	Forest
MTAD1	*Carollia perspicillata*	Male	Adult	Inactive	22	CTAD	Forest
MTAD2	*Carollia perspicillata*	Male	Adult	Inactive	24	CTAD	Forest
MTAD3	*Lonchophylla robusta*	Female	Adult	Inactive	14	CTAD	Forest
MTAE1	*Carollia perspicillata*	Female	Adult	Inactive	16	CTAE	Forest
MTAE3	*Carollia castanea*	Male	Adult	Inactive	10.5	CTAE	Forest
MTAE5	*Glossophaga soricina*	Male	Adult	Inactive	8.5	CTAE	Forest
MTAE7	*Platyrrhinus helleri*	Male	Adult	Inactive	15	CTAE	Forest

Identification	Species	Sex	Age	Reproductive status	Weight (g)	Collection site Identification	Mist Net Location
MTAE9	*Glossophaga soricina*	Male	Adult	Inactive	9.5	CTAE	Forest
MTAE11	*Uroderma convexum*	Male	Adult	Inactive	17	CTAE	Forest
MTAE13	*Glossophaga soricina*	Female	Adult	Inactive	10	CTAE	Forest
MTAE15	*Glossophaga soricina*	Male	Adult	Inactive	9	CTAE	Forest
MTAF1	*Carollia perspicillata*	Male	Adult	Inactive	16	CTAF	Peridomiciliary
MTAF3	*Uroderma convexum*	Male	Adult	Inactive	16	CTAF	Peridomiciliary
MTAF5	*Artibeus lituratus*	Male	Adult	Inactive	58	CTAF	Peridomiciliary
MTAF7	*Uroderma convexum*	Male	Adult	Inactive	17	CTAF	Peridomiciliary
MTAF9	*Carollia perspicillata*	Female	Adult	Pregnant	22.5	CTAF	Peridomiciliary
MTAG1	*Rogheessa io*	Male	Adult	Inactive	4	CTAG	Forest
MTAG3	*Saccopteryx bilineata*	Male	Adult	Inactive	4	CTAG	Forest
MTAG5	*Artibeus jamaicensis*	Female	Adult	Inactive	42	CTAG	Peridomiciliary
MTAG7	*Artibeus jamaicensis*	Female	Adult	Inactive	45	CTAG	Forest
MTAG9	*Artibeus jamaicensis*	Female	Adult	Inactive	53	CTAG	Forest
MTAH1	*Uroderma convexum*	Female	Adult	Pregnant	27	CTAH	Peridomiciliary
MTAH2	*Uroderma convexum*	Female	Adult	Inactive	16	CTAH	Peridomiciliary
MTAH3	*Uroderma convexum*	Male	Adult	Inactive	16	CTAH	Peridomiciliary
MTAH4	*Uroderma convexum*	Female	Adult	Pregnant	22	CTAH	Peridomiciliary
MTAH5	*Uroderma convexum*	Male	Adult	Inactive	16	CTAH	Peridomiciliary
MTAJ1	*Carollia castanea*	Male	Adult	Inactive	13	CTAB	Forest
MTAJ2	*Carollia castanea*	Female	Adult	Pregnant	15	CTAB	River
MTAJ3	*Myotis nigricans*	Female	Adult	Pregnant	5.5	CTAB	River
MTAJ5	*Carollia perspicillata*	Female	Adult	Inactive	20	CTAB	Forest
MTAJ6	*Artibeus jamaicensis*	Male	Adult	Inactive	52	CTAB	Forest

Identification	Species	Sex	Age	Reproductive status	Weight (g)	Collection site Identification	Mist Net Location
MTAJ7	*Artibeus jamaicensis*	Male	Adult	Inactive	46	CTAB	Forest
MTAJ8	*Carollia castanea*	Male	Adult	Inactive	12	CTAB	Peridomiciliary
MTAJ9	*Carollia castanea*	Female	Adult	Inactive	11	CTAB	Peridomiciliary
MTAJ10	*Phyllostomus discolor*	Male	Adult	Inactive	44	CTAB	River
MTAK1	*Artibeus jamaicensis*	Female	Adult	Inactive	54	CTAD	Forest
MTAK3	*Artibeus jamaicensis*	Male	Adult	Inactive	50	CTAD	Forest
MTAK5	*Artibeus jamaicensis*	Male	Adult	Inactive	52	CTAD	Forest
MTAK9	*Artibeus jamaicensis*	Male	Adult	Inactive	45	CTAD	Peridomiciliary
MTAK10	*Rhogeessa io*	Female	Adult	Inactive	3.5	CTAD	Hen House
MTAL1	*Artibeus jamaicensis*	Male	Adult	Inactive	45	CTAC	Peridomiciliary
MTAL2	*Artibeus jamaicensis*	Female	Adult	Inactive	44	CTAC	Forest
MTAL3	*Glossophaga soricina*	Male	Adult	Inactive	10.5	CTAC	Forest
MTAM1	*Carollia perspicillata*	Female	Adult	Inactive	20	CTAF	Peridomiciliary
MTAM3	*Carollia perspicillata*	Male	Adult	Inactive	22	CTAF	Forest
MTAM5	*Carollia perspicillata*	Male	Adult	Inactive	20	CTAF	Forest
MTAM7	*Artibeus jamaicensis*	Female	Adult	Inactive	39	CTAF	Peridomiciliary
MTAM9	*Carollia perspicillata*	Male	Adult	Inactive	21	CTAF	Forest
MTAN1	*Glossophaga soricina*	Female	Adult	Inactive	10	CTAE	Forest
MTAN3	*Artibeus jamaicensis*	Female	Adult	Inactive	56	CTAE	Forest
MTAN5	*Uroderma convexum*	Male	Adult	Inactive	18	CTAE	Forest
MTAN7	*Carollia castanea*	Female	Adult	Inactive	12.5	CTAE	Peridomiciliary
MTAN9	*Carollia castanea*	Female	Adult	Inactive	13	CTAE	Peridomiciliary
MTAO1	*Rhogeessa io*	Female	Adult	Inactive	4.5	CTAH	Peridomiciliary
MTAO3	*Uroderma convexum*	Female	Adult	Inactive	16	CTAH	Peridomiciliary

Identification	Species	Sex	Age	Reproductive status	Weight (g)	Collection site Identification	Mist Net Location
MTAO5	*Uroderma convexum*	Male	Adult	Inactive	16.5	CTAH	Peridomiciliary
MTAO7	*Uroderma convexum*	Male	Adult	Inactive	17.5	CTAH	Peridomiciliary
MTAO9	*Carollia perspicillata*	Female	Adult	Inactive	20.5	CTAH	Forest
