# Supplementary material for: Serological Positivity against Selected Flaviviruses and Alphaviruses in Free-Ranging Bats and Birds from Costa Rica Evidence Exposure to Arboviruses Seldom Reported Locally in Humans
Source: Viruses. 2022 Jan 6;14(1):93. doi: 10.3390/v14010093 (PMC8780000; doi:10.3390/v14010093)
Supplement: Supplementary file 1 [file viruses-14-00093-s001.zip › Supplementary Table S3.pdf]

Supplementary Table S3. List of birds collected in Santa Cruz.

**Identification**	**Species**	**Sex**	**Age**	**Reproductive status**	**Weight (g)**	**Collection site Identification**	**Mist Net Location**
ASCA1	*Aimophila ruficauda*	Male	Adult	Inactive	37	CSCA	Peridomiciliary
ASCA2	*Aimophila ruficauda*	Male	Adult	Inactive	40	CSCA	Peridomiciliary
ASCA3	*Amazilia rutila*	Na	Adult	Inactive	10	CSCA	Peridomiciliary
ASCA4	*Aimophila ruficauda*	Male	Adult	Inactive	36	CSCA	Peridomiciliary
ASCA5	*Euphonia hirundinacea*	Male	Adult	Inactive	17	CSCA	Peridomiciliary
ASCA6	*Columbina inca*	NA	Adult	NA	NA	CSCA	Peridomiciliary
ASCB1	*Pitangus sulphuratus*	Male	Adult	Inactive	80	CSCB	Peridomiciliary
ASCC1	*Polioptila plúmbea*	Male	Adult	Inactive	7	CSCC	Forest
ASCC2	*Columbina inca*	Female	Adult	Inactive	54	CSCC	Forest
ASCD1	*Turdus grayi*	Male	Adult	Inactive	82	CSCD	Forest
ASCD2	*Turdus grayi*	Male	Adult	Inactive	80	CSCD	Forest
ASCE1	*Amazilia rutila*	Female	Adult	Inactive	4	CSCE	Forest
ASCE2	*Amazilia rutila*	Female	Adult	Inactive	4	CSCE	Forest
ASCH1	*Campylorhynchus rufinucha*	Male	Adult	Inactive	28	CSCH	Peridomiciliary 1
ASCH2	*Campylorhynchus rufinucha*	Male	Adult	Inactive	29	CSCH	Peridomiciliary 1
ASCH3	*Campylorhynchus rufinucha*	Male	Adult	Inactive	35	CSCH	Peridomiciliary 1
ASCI1	*Eumomota superciliosa*	Female	Adult	Inactive	57	CSCA	River
ASCI2	*Columbina inca*	Female	Adult	Inactive	36	CSCA	River 2
ASCI3	*Columbina inca*	Male	Adult	Inactive	52	CSCA	River 2
ASCI4	*Columbina inca*	Female	Adult	Inactive	38	CSCA	River 1
ASCI5	*Quiscalus mexicanus*	Female	Adult	Inactive	120	CSCA	River 1
ASCI6	*Pitangus sulphuratus*	Female	Adult	Inactive	65	CSCA	Forest

Identification	Species	Sex	Age	Reproductive status	Weight (g)	Collection site Identification	Mist Net Location
ASCI7	*Pitangus sulphuratus*	Female	Adult	Inactive	64	CSCA	Forest
ASCJ1	*Columbina inca*	Female	Adult	Inactive	52	CSCB	Barn 1
ASCJ2	*Turdus grayi*	Male	Adult	Inactive	68	CSCB	Barn
ASCJ3	*Columbina inca*	Female	Adult	Inactive	37.5	CSCB	Barn
ASCK1	*Pitangus sulphuratus*	Female	Adult	Inactive	75	CSCC	Net 1
ASCK2	*Quiscalus mexicanus*	Female	Adult	Inactive	115	CSCC	Net 3
ASCK3	*Amazilia rutila*	Male	Adult	Inactive	5	CSCC	Net 2
ASCK4	*Quiscalus mexicanus*	Male	Adult	Inactive	115	CSCC	Net 2
ASCK5	*Campylorhynchus rufinucha*	Male	Adult	Inactive	29	CSCC	Net 2
ASCL1	*Campylorhynchus rufinucha*	Female	Adult	Inactive	29	CSCD	Net Hill
ASCL2	*Columbina inca*	Male	Adult	Inactive	50	CSCD	Peridomiciliary
ASCL3	*Columbina inca*	NA	Adult	Inactive	60	CSCD	Peridomiciliary
ASCM1	*Columbina inca*	Female	Adult	Inactive	61	CSCE	Peridomiciliary
ASCM2	*Columbina inca*	Male	Adult	Inactive	49	CSCE	Hill
ASCM3	*Columbina inca*	Male	Adult	Inactive	53	CSCE	Forest
ASCM4	*Columbina inca*	Female	Adult	Inactive	52	CSCE	Hill
ASCM5	*Icterus pustulatus*	Male	Adult	Inactive	45	CSCE	Hill
ASCM6	*Icterus pustulatus*	Male	Adult	Inactive	40	CSCE	Hill
ASCM7	*Vireo flavoviridis*	Male	Adult	Inactive	16	CSCE	Hill
ASCM8	*Icterus pustulatus*	Male	Adult	Inactive	45	CSCE	Hill
ASCM9	*Campylorhynchus rufinucha*	Female	Adult	Inactive	33.5	CSCE	Hill
ASCM10	*Vireo flavoviridis*	Female	Adult	Inactive	26	CSCE	Hill
ASCM11	No identificado	Male	Adult	Inactive	51	CSCE	Hill
ASCM12	*Geothlypis philadelphia*	Male	Adult	Inactive	8	CSCE	Forest

Identification	Species	Sex	Age	Reproductive status	Weight (g)	Collection site Identification	Mist Net Location
ASCN1	*Amazilia rutila*	Male	Adult	Inactive	4.5	CSCF	Forest
ASCN2	*Tolmomyias sulphurescens*	Female	Adult	Inactive	14.5	CSCF	Forest
ASCN3	*Tolmomyias sulphurescens*	Female	Adult	Inactive	16	CSCF	Forest
ASCN4	*Campylorhynchus rufinucha*	Male	Adult	Inactive	35	CSCF	Forest
ASCN5	*Tolmomyias sulphurescens*	Male	Adult	Inactive	19	CSCF	Forest
ASCN6	*Thryophilus pleurostictus*	Male	Adult	Inactive	16.5	CSCF	Forest
