# Supplementary material for: Serological Positivity against Selected Flaviviruses and Alphaviruses in Free-Ranging Bats and Birds from Costa Rica Evidence Exposure to Arboviruses Seldom Reported Locally in Humans
Source: Viruses. 2022 Jan 6;14(1):93. doi: 10.3390/v14010093 (PMC8780000; doi:10.3390/v14010093)
Supplement: Supplementary file 1 [file viruses-14-00093-s001.zip › Supplementary Table S4.pdf]

Supplementary Table S4. List of birds collected in Talamanca.

Identification	Species	Sex	Age	Reproductive status	Weight (g)	Collection site Identification	Mist Net Location
ATAA1	*Amazilia tzacatl*	Female	Adult	Inactive	6	CTAA	Peridomiciliary
ATAA2	*Sporophila morelleti*	Male	Adult	Inactive	13	CTAA	Barn
ATAA3	*Sporophila morelleti*	Male	Adult	Inactive	11	CTAA	Forest
ATAA4	*Pitangus sulphuratus*	Male	Adult	Inactive	64	CTAA	Forest
ATAA5	*Pitangus sulphuratus*	Male	Adult	Inactive	60	CTAA	Forest
ATAA6	*Sporophila morelleti*	Male	Adult	Inactive	11	CTAA	Barn
ATAA7	*Amazilia tzacatl*	Female	Adult	Inactive	5	CTAA	Barn
ATAA8	*Crotophaga sulcirostris*	Male	Adult	Inactive	77	CTAA	Forest
ATAA9	*Ramphocelus passerinii*	Male	Adult	Inactive	20	CTAA	Forest
ATAA10	*Sporophila morelleti*	Female	Adult	Inactive	10	CTAA	Forest
ATAA11	*Sporophila morelleti*	Male	Adult	Inactive	10	CTAA	Forest
ATAA12	*Tyrannus melancholicus*	Male	Adult	Inactive	25	CTAA	Forest
ATAA13	*Sporophila morelleti*	Male	Adult	Inactive	11.5	CTAA	Forest
ATAA14	*Sporophila morelleti*	Male	Adult	Inactive	10	CTAA	Forest
ATAA15	*Sporophila morelleti*	Male	Adult	Inactive	10	CTAA	Forest
ATAA16	*Sporophila morelleti*	Female	Adult	Inactive	10.5	CTAA	Forest
ATAA17	*Sporophila morelleti*	Male	Adult	Inactive	10	CTAA	Forest
ATAA18	*Sporophila morelleti*	Male	Adult	Inactive	10	CTAA	Forest
ATAA19	*Turdus grayi*	Female	Adult	Inactive	70	CTAA	Forest
ATAA20	*Nyctidromus albicollis*	Female	Adult	Inactive	56	CTAA	Forest
ATAA21	*Tyrannus melancholicus*	Male	Adult	Inactive	38	CTAA	Forest
ATAA22	*Ramphocelus passerinii*	Male	Adult	Inactive	28	CTAA	Forest

Identification	Species	Sex	Age	Reproductive status	Weight (g)	Collection site Identification	Mist Net Location
ATAB1	*Amazilia tzacatl*	Male	Adult	Inactive	5	CTAB	Forest
ATAB2	*Glaucis aeneus*	Female	Adult	Inactive	6	CTAB	Forest
ATAB3	*Turdus grayi*	Male	Adult	Inactive	70	CTAB	Forest
ATAC1	*Amazilia tzacatl*	Female	Adult	Inactive	7	CTAC	Forest 1
ATAC2	*Habia fuscicauda*	Male	Adult	Inactive	47	CTAC	Forest 2
ATAC3	*Phaethornis striigularis*	Male	Adult	Inactive	2.5	CTAC	Peridomiciliary
ATAC4	*Habia fuscicauda*	Female	Adult	Inactive	28	CTAC	Peridomiciliary
ATAC5	*Habia fuscicauda*	Female	Adult	Inactive	39.5	CTAC	Forest 2
ATAC6	*Chloroceryle amazona*	Male	Adult	Inactive	32	CTAC	Forest 2
ATAC7	*Amazilia tzacatl*	Female	Adult	Inactive	4.5	CTAC	Forest 2
ATAC8	*Xiphorhynchus erythropygius*	Female	Adult	Inactive	43	CTAC	Forest 2
ATAD1	*Glaucis aeneus*	Male	Adult	Inactive	7	CTAD	Peridomiciliary
ATAD2	*Glaucis aeneus*	Male	Adult	Inactive	7	CTAD	Peridomiciliary
ATAD3	*Amazilia tzacatl*	Male	Adult	Inactive	5	CTAD	Peridomiciliary
ATAE1	*Phaethornis longirostris*	Male	Adult	Inactive	6	CTAE	Forest
ATAE2	*Troglodytes aedon*	Male	Adult	Inactive	10	CTAE	Paddock
ATAE3	*Glaucis aeneus*	Male	Adult	Inactive	5	CTAE	Forest
ATAE4	*Sporophila morelleti*	Female	Adult	Inactive	10	CTAE	Paddock
ATAE5	*Sporophila funérea*	Female	Adult	Inactive	12	CTAE	Paddock
ATAE6	*Sporophila morelleti*	Female	Adult	Inactive	10	CTAE	Paddock
ATAE7	*Todirostrum cinereum*	Male	Adult	Inactive	5.5	CTAE	Paddock
ATAE8	*Sporophila morelleti*	Male	Adult	Inactive	9.5	CTAE	Paddock
ATAE9	*Sporophila morelleti*	Male	Adult	Inactive	10.5	CTAE	Paddock
ATAE10	*Dendrocincla fuliginosa*	Male	Adult	Inactive	37	CTAE	Forest

Identification	Species	Sex	Age	Reproductive status	Weight (g)	Collection site Identification	Mist Net Location
ATAE11	*Sporophila morelleti*	Female	Adult	Inactive	10	CTAE	Forest
ATAE12	*Sporophila morelleti*	Female	Adult	Inactive	9.5	CTAE	Forest
ATAE13	*Mionectes oleagineus*	Male	Adult	Inactive	10.5	CTAE	Forest
ATAF1	*Myiozetetes similis*	Female	Adult	Inactive	20.5	CTAF	Peridomiciliary
ATAF2	*Euphonia luteicapilla*	Male	Adult	Inactive	10.5	CTAF	Forest
ATAF3	*Phaethornis longirostris*	Male	Adult	Inactive	6	CTAF	Peridomiciliary
ATAF4	*Crotophaga sulcirostris*	Male	Adult	Inactive	83	CTAF	Forest
ATAF5	*Troglodytes aedon*	Female	Adult	Inactive	11	CTAF	Peridomiciliary
ATAH1	*Myiozetetes similis*	Male	Adult	Inactive	26	CTAH	Forest
ATAH2	*Myiozetetes similis*	Male	Adult	Inactive	27	CTAH	Forest
ATAH3	*Myiozetetes similis*	Male	Adult	Inactive	29	CTAH	Forest
ATAH4	*Myiozetetes similis*	Male	Adult	Inactive	29	CTAH	Forest
ATAH5	*Myiozetetes similis*	Male	Adult	Inactive	25	CTAH	Forest
ATAH6	*Troglodytes aedon*	Male	Adult	Inactive	13	CTAH	Forest
ATAI1	*Patagioenas flavirostris*	Male	Adult	Inactive	260	CTAA	Forest
ATAI2	*Sporophila morelleti*	Male	Adult	Inactive	11.5	CTAA	Forest
ATAI3	*Sporophila morelleti*	Male	Adult	Inactive	9.5	CTAA	Forest
ATAI4	*Sporophila morelleti*	Female	Adult	Inactive	10.5	CTAA	Forest
ATAI5	*Sporophila morelleti*	Male	Adult	Inactive	11	CTAA	Forest
ATAI6	*Pitangus sulphuratus*	Female	Adult	Inactive	60	CTAA	Peridomiciliary
ATAI7	*Pitangus sulphuratus*	Female	Adult	Inactive	63	CTAA	Peridomiciliary
ATAI8	*Sporophila morelleti*	NA	Adult	Inactive	10	CTAA	Forest
ATAJ1	*Empidonax virescens*	Male	Adult	Inactive	14.5	CTAB	Peridomiciliary
ATAJ2	*Catharus ustulatus*	Male	Adult	Inactive	27	CTAB	Forest

Identification	Species	Sex	Age	Reproductive status	Weight (g)	Collection site Identification	Mist Net Location
ATAJ3	*Empidonax virescens*	Male	Adult	Inactive	12	CTAB	Forest
ATAJ4	*Xiphorhynchus susurrans*	Female	Adult	Inactive	46.5	CTAB	Forest
ATAK1	*Phaethornis striigularis*	Female	Adult	Inactive	2.5	CTAD	Forest
ATAL1	*Turdus grayi*	Female	Adult	Inactive	79	CTAC	Peridomiciliary
ATAL2	*Parkesia noveboracensis*	Male	Adult	Inactive	14	CTAC	Peridomiciliary
ATAL3	*Empidonax alnorum*	Male	Adult	Inactive	12.5	CTAC	Peridomiciliary
ATAL4	*Piranga rubra*	Male	Adult	Inactive	32	CTAC	Forest
ATAL5	*Empidonax virescens*	Male	Adult	Inactive	10	CTAC	Forest
ATAM1	*Empidonax virescens*	Male	Adult	Inactive	13.5	CTAF	Peridomiciliary
ATAN1	*Amazilia tzacatl*	Male	Adult	Inactive	5	CTAE	Forest
ATAN2	*Crotophaga sulcirostris*	Female	Adult	Inactive	73	CTAE	Peridomiciliary
ATAN3	*Crotophaga sulcirostris*	Male	Adult	Inactive	81	CTAE	Peridomiciliary
ATAN4	*Dendrocincla fuliginosa*	Male	Adult	Inactive	46	CTAE	Peridomiciliary
ATA01	*Vireo flavifrons*	Male	Adult	Inactive	16	CTAH	Forest
ATA02	*Amazilia tzacatl*	Male	Adult	Inactive	6	CTAH	Peridomiciliary
ATA03	*Sporophila morelleti*	Male	Adult	Inactive	11	CTAH	Forest
ATA04	*Myiozetetes similis*	Male	Adult	Inactive	27	CTAH	Forest
ATA05	*Parkesia noveboracensis*	Male	Adult	Inactive	14.5	CTAH	Forest
